# Comparison of 0.1% Ropivacaine and 0.0625% Bupivacaine With Fentanyl as an Adjuvant for Labour Analgesia

**DOI:** 10.7759/cureus.112235

**Published:** 2026-07-07

**Authors:** Ranganaayaki Ganessane, Krishna Prabu, Mannar Mannan, Balapriya Boopathy

**Affiliations:** 1 Department of Anaesthesiology, Aarupadai Veedu Medical College and Hospital, Puducherry, IND; 2 Department of Anaesthesiology, Sri Venkateshwaraa Medical College Hospital and Research Centre, Puducherry, IND; 3 Department of Anesthesiology, Vinayaka Missions Medical College and Hospital, Karaikal, IND; 4 Department of Anaesthesiology, Sri Manakula Vinayagar Medical College and Hospital, Puducherry, IND

**Keywords:** bupivacaine, epidural analgesia, fentanyl, labour analgesia, ropivacaine

## Abstract

Background and aims

Effective pain relief during labour is an important component of obstetric care, with epidural analgesia being the most reliable method. Using low concentrations of local anaesthetics combined with opioids can provide adequate analgesia while minimizing adverse effects. In this study, we compared the analgesic effectiveness of 0.1% ropivacaine and 0.0625% bupivacaine, each administered with fentanyl (2 mcg/mL), for epidural labour analgesia.

Methods

A double-blinded randomized controlled study was conducted with 40 parturients belonging to ASA physical status I or II. They were divided into two groups of 20 each by computer-generated block randomization. Group A received bolus doses of 0.0625% bupivacaine with 2 mcg/mL fentanyl. Group B received bolus doses of 0.1% ropivacaine with 2 mcg/mL fentanyl. The outcomes assessed included hemodynamic stability, sensory blockade, motor blockade, pain score using a visual analogue scale (VAS), number of supplemental doses, total drug consumption, first and second stages of labour duration, mode of delivery, maternal satisfaction, outcomes of the newborn, and adverse effects such as hypotension, bradycardia, nausea, and vomiting.

Results

The sensory blockade onset was significantly faster in Group A compared to Group B (p = 0.03). VAS scores were significantly lower in Group A at 5 minutes (p < 0.0001), 10 minutes (p = 0.0003), 15 minutes (p = 0.02), 1 hour (p < 0.001), and 2 hours (p = 0.0002). Group A also required fewer additional doses and demonstrated lower overall drug consumption (p = 0.02). Maternal satisfaction scores were significantly higher in Group A (p = 0.01).

Conclusion

Epidural labour analgesia with 0.0625% bupivacaine with fentanyl (2 mcg/mL) provides superior analgesic efficacy, faster onset, and reduced drug requirements compared to 0.1% ropivacaine with fentanyl (2 mcg/mL), while maintaining a comparable safety profile.

## Introduction

Labour pain is one of the most severe pains experienced by women [1]. According to the American College of Obstetricians and Gynaecologists and the American Society of Anaesthesiologists, effective pain relief should be offered to all parturients, and maternal request alone constitutes a valid indication for labour analgesia in the absence of contraindications [2].

The physiological stress response to labour pain leads to sympathetic nervous system activation, increased cardiac output, and hypertension. These changes may reduce uteroplacental perfusion and can adversely influence the progress of labour [3-5]. In addition to physiological effects, inadequately managed labour pain has been associated with negative psychological outcomes, including impaired maternal-infant bonding and postpartum mood disturbances [6].

An ideal labour analgesic technique should provide consistent and effective pain relief without compromising maternal safety, fetal well-being, or the natural progression of labour [7]. Among the available options, neuraxial techniques, particularly epidural analgesia, are considered the most reliable method for achieving effective analgesia during labour. These techniques offer superior pain control without significant maternal or neonatal sedation [8].

The combination of low concentrations of local anaesthetics with lipid-soluble opioids enhances analgesic efficacy while minimising dose-related adverse effects. Agents such as fentanyl are frequently used as adjuvants in epidural analgesia due to their rapid onset and synergistic action with local anaesthetics [9]. Epidural analgesia also offers the advantage of flexibility, as it can be extended to provide surgical anaesthesia if operative delivery becomes necessary [10,11]. A safe alternative to bupivacaine was considered to be ropivacaine for labour analgesia [12,13]. 

Previous studies have evaluated various concentrations of bupivacaine (0.0625-0.125%) and ropivacaine (0.1-0.125%) for labour analgesia, with differing results regarding efficacy and side effect profiles, including motor blockade and hemodynamic instability [12,14,15].

In this study, the primary outcome was to compare the analgesic efficacy of 0.0625% bupivacaine versus 0.1% ropivacaine, both combined with fentanyl (2 mcg/mL), in providing epidural labour analgesia using the VAS score. The secondary outcome was to compare the hemodynamic stability, sensory and motor blockade, onset of analgesia, total amount of drug required, and number of top-ups, first and second stages of labour duration, mode of delivery, outcomes of newborn, and adverse effects such as hypotension, bradycardia, nausea, and vomiting.

## Materials and methods

This prospective, double-blind, randomized controlled study was conducted at a tertiary care centre after obtaining approval from the Institutional Ethics Committee (Approval No: outward/SMVMCH-ECO/AL/146/2018). The study was registered with the Clinical Trials Registry of India (CTRI/2019/05/019271) and adhered to Good Clinical Practice guidelines [16]. A total of 40 parturients with American Society of Anaesthesiologists (ASA) physical status I or II, carrying term singleton pregnancies and requesting epidural labour analgesia, were enrolled. Written informed consent was obtained prior to inclusion (Table 1).

**Table 1:** Inclusion and Exclusion criteria

Inclusion criteria	Exclusion criteria
Parturients who requested epidural analgesia	Age < 18 years
Parturients of ASA physical status 1 & 2	Cognitive impairment
Parturients in active labour with cervical dilation of > 3 cm	Contraindications for epidural anaesthesia
Parturients with full-term live fetus	Drug hypersensitivity
Parturients without any obstetric complications	Needed IV analgesics after epidural administration

The sample size was estimated based on the primary outcome of analgesic efficacy assessed with a visual analogue scale (VAS) as used in a study by Chethanananda et al. The study compared 0.0625% bupivacaine with fentanyl and 0.1% ropivacaine with fentanyl for epidural labour analgesia; a clinically significant difference between the study groups was anticipated [14]. Considering a two-sided significance level of 5% and a study power of 80%, the minimum required sample size was calculated as 20 participants per group. Therefore, a total of 40 parturients were enrolled and randomly allocated into two groups of 20 participants each.

**Figure 1:**
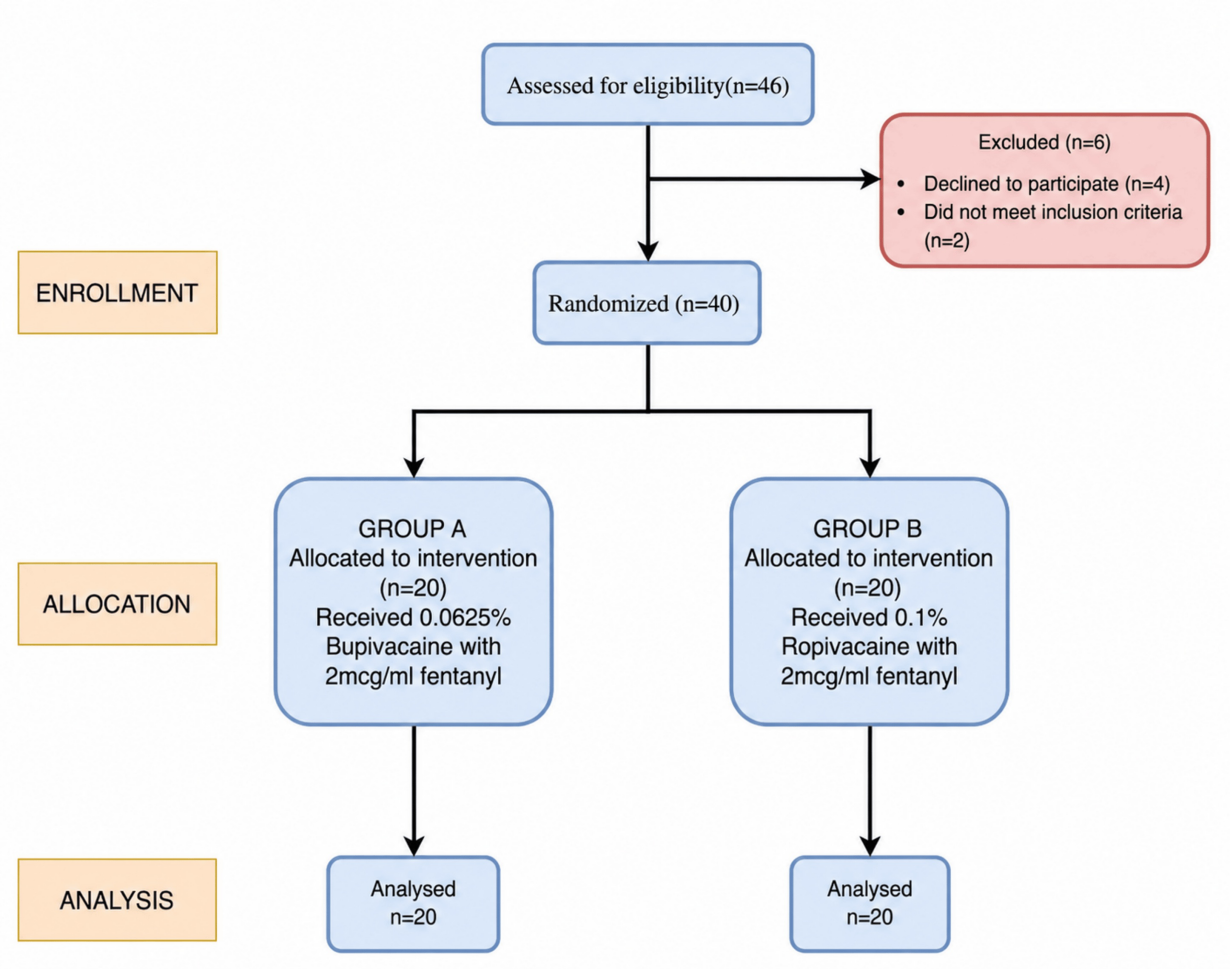
CONSORT Flow Diagram of the study CONSORT [17]

Participants were randomly allocated into two groups (n=20 each) using a computer-generated block randomisation method with allocation concealment ensured via sealed opaque envelopes. Group A received bolus doses of 0.0625% bupivacaine with fentanyl 2 mcg/mL. Group B received bolus doses of 0.1% ropivacaine with fentanyl 2 mcg/mL (Figure 1).

The drug was prepared by an anaesthesiologist not involved in the study to maintain blinding. Both the investigator and the data recorder were unaware of group allocation. Standard monitoring, including non-invasive blood pressure, pulse oximetry, and electrocardiography, was connected. Hemodynamic parameters were recorded throughout the study. Parturients were preloaded with 500 mL of Ringer's lactate solution, and intermittent sips of clear fluids were allowed. Injection ranitidine 50 mg and injection metoclopramide 10mg were given intravenously for anti-aspiration prophylaxis. 

Under aseptic precautions, the epidural space was identified at the L3-L4 intervertebral space using the loss-of-resistance technique, and an epidural catheter was inserted. A test dose of 3 mL of 1.5% lignocaine containing epinephrine 5 μg/mL was administered to exclude intrathecal or intravascular catheter placement. Subsequently, a 10 mL bolus of the study solution was administered through the epidural catheter.

Sensory blockade was assessed using loss of cold sensation along the mid-clavicular line. The onset of sensory block up to T10 level and the highest sensory level achieved were recorded. Sensory block was assessed every minute until onset, every 5 minutes for the first 30 minutes, at 1 hour, at 2 hours, and every 2 hours thereafter until delivery. Motor blockade was evaluated using the modified Bromage scale.

Pain intensity was assessed using the VAS during peak uterine contractions. VAS scores were recorded before epidural administration, every 5 minutes for the first 30 minutes, at 1 hour, at 2 hours, and every 2 hours thereafter until delivery. 

If the VAS score remained ≥4 at 15 minutes after the initial epidural bolus, a 5 mL bolus of the study solution was administered at intervals of 5 minutes, up to a maximum cumulative volume of 20 mL. Epidural analgesia was considered inadequate if the VAS score remained ≥4 despite administration of a total of 20 mL of study solution. Such parturients received alternative analgesia with intravenous pethidine in 10 mg increments and were excluded from further analysis.

During labour, whenever the VAS score was ≥4, an epidural top-up of 5 mL of the study solution was administered, maintaining a minimum interval of 5 minutes between doses and a maximum hourly dose of 20 mL.

The total amount of study drug administered (mL) and the number of epidural top-ups required were recorded. The duration of the first and second stages of labour, mode of delivery, and neonatal outcome assessed using APGAR scores at 1 and 5 minutes were documented. Maternal adverse effects, including hypotension, nausea, vomiting, and respiratory depression, were monitored throughout the study period.

After delivery, parturients were asked to rate their satisfaction score as either excellent, good, fair, or poor to know the quality of analgesia.

Statistical analysis

All the statistical tests were done using SPSS version 20 (IBM Corp., Armonk, NY). The normality of the data was analysed using the Shapiro-Wilk test. Categorical data were expressed as frequencies and percentages, whereas continuous data were expressed as mean ± SD. Continuous variables, including age and anthropometry, VAS pain scores, number of top-ups required, total drug requirement, and duration of labour, were compared using Student's independent t-test. The Wilcoxon rank-sum test was used for VAS pain scores when appropriate. Gender, ASA grade, and type of procedure were compared using the chi-square test. Hemodynamic parameters such as heart rate, systolic blood pressure, diastolic blood pressure, and mean arterial pressure were analysed using repeated measures analysis of variance (ANOVA). Effect size for continuous variables was quantified using Cohen's d, with values of 0.2, 0.5, and 0.8 representing small, moderate, and large effects, respectively. Degrees of freedom were reported for categorical variables analysed using the chi-square or Fisher's exact test. A p-value of < 0.05 was considered significant.

## Results

Baseline characteristics of parturients included in the study are shown in Table 2. There was no statistically significant difference between the groups with a p-value > 0.05.

**Table 2:** Demographic and Baseline Characteristics of the Study Population Data are presented as mean ± SD or number (%). *P-value calculated using Student’s t-test for continuous variables and chi-square test for categorical variables.

Variable	Group A (n=20)	Group B (n=20)	P-value*
Age (years)	25.15 ± 3.30	26.30 ± 3.37	0.90
Height (cm)	157.45 ± 2.78	156.05 ± 4.02	0.21
Weight (kg)	74.15 ± 8.21	75.80 ± 6.89	0.49
BMI (kg/m²)	29.89 ± 2.75	31.17 ± 2.87	0.16
Gestational age (weeks)	38.75 ± 0.99	38.83 ± 1.04	0.79
Parity			0.75
Primi gravida	11 (55%)	10 (50%)	
Multigravida	9 (45%)	10 (50%)	
Cervical dilatation (cm)			0.38
3 cm	1 (5%)	0 (0%)	
4 cm	15 (75%)	13 (65%)	
5 cm	4 (20%)	7 (35%)	

**Figure 2:**
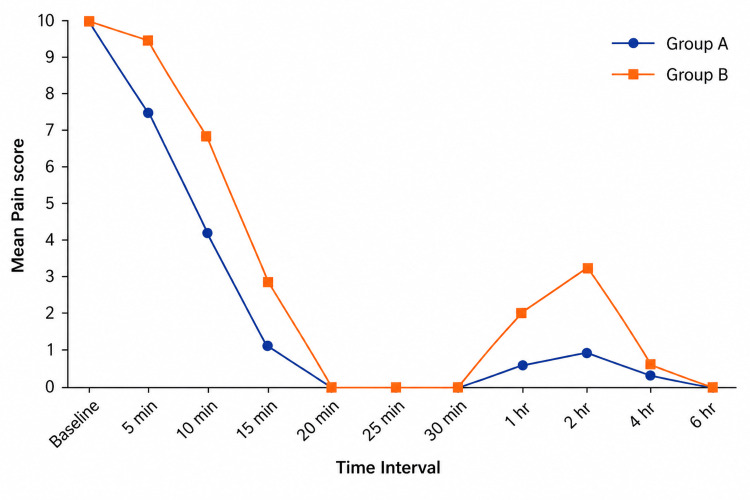
Distribution of pain score across different time intervals

Table 3 and Figure 2 show that the baseline pain score was found to be 10 in both groups according to the VAS score. Following the epidural injection of the study drug, the pain score was reduced in both groups. Pain score assessed by the VAS was found to be less in Group A compared to Group B at 5 min with a p-value of <0.0001, at 10 min with a p-value of 0.0003, at 15 min with a p-value of 0.02, at 1 hour with a p-value of <0.001, and at 2 hours with a p-value of 0.0002. 

**Table 3:** Distribution of pain score across different time intervals *p-value by Student’s t-test ǂP value by the Wilcoxon Rank sum test

VAS Score	Group A (n=20)		Group B (n=20)		p-value*
	Mean	SD	Mean	SD	
At baseline	10	0	10	0	1
5 mins	7.60	1.35	9.55	0.76	<0.0001
10 minsǂ	4.2	1.77	6.8	2.28	0.0003
15 minsǂ	1.1	1.55	2.85	2.81	0.02
20 mins	0	0	0	0	1
25 mins	0	0	0	0	1
30 mins	0	0	0	0	1
1 hrǂ	0.6	0.9	2.05	1.1	<0.001
2 hrsǂ	0.85	1.39	3.2	2.14	0.0002
4 hrsǂ	0.43	1.16	0.41	0.94	0.85
6 hrs (A:14 B:17)	0	0	0	0	1

The onset of sensory block was 13.45 ± 3.66 minutes in Group A and 16.00 ± 3.48 minutes in Group B, which was statistically significant (p = 0.03). The effect size was moderate to large (Cohen's d = 0.71) (Table 4).

**Table 4:** Comparison of onset of sensory block up to T10 level *p-value by Student’s t-test

Variable	Group A (n=20) Mean ± SD	Group B (n=20) Mean ± SD	Effect Size (Cohen's d)	p-value*
Onset of sensory block up to T10 level (min)	13.45 ± 3.66	16.00 ± 3.48	0.71	0.03

The highest sensory block level was comparable between the two groups and was not statistically significant (Fisher's exact test, df = 3, p = 0.84) (Table 5).

**Table 5:** Comparison of the highest sensory block level *p-value by Fisher's exact chi-square test

Sensory Block Level	Group A (n=20) n (%)	Group B (n=20) n (%)	Total	df	p-value*
T7	5 (25.0)	5 (25.0)	10	3	0.84
T8	10 (50.0)	12 (60.0)	22		
T9	1 (5.0)	1 (5.0)	2		
T10	4 (20.0)	2 (10.0)	6		
Total	20 (100.0)	20 (100.0)	40		

The number of top-ups in Group A was fewer than in Group B. The difference was statistically significant (p = 0.02), with a moderate to large effect size (Cohen's d = 0.78) (Table 6).

**Table 6:** Distribution of the number of top-ups * p-value by Student's t-test

Group	Mean ± SD	Effect Size (Cohen's d)	p-value*
A (n=20)	3.30 ± 1.03	0.78	0.02
B (n=20)	4.15 ± 1.14		

The total drug requirement (mL) was lower in Group A than in Group B. The difference was statistically significant (p = 0.02), with a large effect size (Cohen's d = 0.81) (Table 7).

**Table 7:** Distribution of total drug requirements (mL) between the groups * p-value by Student's t-test

Group	Mean ± SD	Effect Size (Cohen's d)	p-value*
A (n=20)	26.5 ± 5.16	0.81	0.02
B (n=20)	31.0 ± 5.98		

Parturient satisfaction was significantly better in Group A than in Group B (χ² = 6.67, df = 1, p = 0.01) (Table 8).

**Table 8:** Comparison of parturient satisfaction *p-value by chi-square test

Parturient Satisfaction	Group A (n=20) n (%)	Group B (n=20) n (%)	χ²	df	p-value*
Good	8 (40.0)	16 (80.0)	6.67	1	0.01
Excellent	12 (60.0)	4 (20.0)			
Total	20 (100.0)	20 (100.0)			

No motor blockade was observed in either group. The durations of the first and second stages of labour were comparable between the two groups and found to be statistically insignificant with a p-value > 0.05. Three parturients in Group A and two parturients in Group B were delivered by lower segment caesarean section. The mode of delivery was found to be statistically insignificant with a p-value > 0.05. Both groups were comparable in terms of APGAR score at 1 min and at 5 min; no significant difference was observed between the groups with a p-value > 0.05. 

The overall between-group effect across all time points was not statistically significant (repeated measures ANOVA p = 0.77), indicating that heart rate profiles over the study period were comparable between the two epidural drug regimens. None of the individual time-point comparisons reached statistical significance, consistent with the omnibus test result (Table 9).

**Table 9:** Heart rate (/min) at different time points between the groups To account for the correlation structure of repeated measurements on the same patients across 14 time points (baseline through 24 hours), a two-factor repeated measures analysis of variance (ANOVA) was performed. In this model, group (0.0625% bupivacaine + fentanyl vs 0.1% ropivacaine + fentanyl) was treated as the between-subjects factor, and time point was treated as the within-subjects factor; each patient contributed one observation per time point, and the model partitioned the total variance into components attributable to group, time, and their interaction. Repeated measures ANOVA: p = 0.77.

Time point	0.0625% bupivacaine + fentanyl (n=20); mean (SD)	0.1% ropivacaine + fentanyl (n=20); mean (SD)
Baseline	88.7 (10.77)	90.2 (6.82)
5 min	85.6 (10.02)	85.5 (7.39)
10 min	82.1 (8.68)	81.8 (6.91)
15 min	80.9 (8.59)	80.0 (6.95)
20 min	80.1 (8.62)	79.5 (7.37)
25 min	81.0 (7.12)	79.4 (6.80)
30 min	80.1 (7.14)	79.0 (6.94)
1 hour	80.5 (6.98)	79.5 (5.22)
2 hours	81.3 (8.67)	80.7 (5.86)
4 hours	81.2 (7.71)	78.8 (4.91)
6 hours	79.0 (5.00)	79.8 (4.65)
8 hours	79.6 (4.78)	78.0 (3.04)
12 hours	78.3 (5.12)	79.8 (5.05)
24 hours	80.0 (4.96)	79.6 (4.85)

Repeated measures ANOVA was applied to compare systolic blood pressure over time between the groups. The overall between-group effect was non-significant (p = 0.46), indicating that systolic blood pressure trends were statistically comparable between the 0.0625% bupivacaine + fentanyl and 0.1% ropivacaine + fentanyl groups throughout the observation period. The small mean differences observed at individual time points did not reach clinical or statistical significance (Table 10). 

**Table 10:** Systolic blood pressure (mmHg) at different time points between the groups To examine the overall trajectory of systolic blood pressure across all 14 time points simultaneously and to account for the within-patient correlation inherent in serial measurements, a repeated measures ANOVA was applied. The model incorporated drug group as the between-subjects factor--with each of the 40 patients assigned exclusively to one group--and time point as the within-subjects factor, with each patient contributing 14 repeated observations. This design allows the F-test for the group effect to control for individual patient variability, yielding a more powerful and appropriate test of sustained group differences. Repeated measures ANOVA: p = 0.46.

Time point	0.0625% bupivacaine + fentanyl (n=20); mean (SD)	0.1% ropivacaine + fentanyl (n=20); mean (SD)
Baseline	126.7 (5.88)	129.5 (5.15)
5 min	119.7 (9.94)	123.9 (6.70)
10 min	116.2 (9.57)	119.9 (6.34)
15 min	115.8 (8.75)	118.1 (6.92)
20 min	114.7 (7.54)	117.2 (4.01)
25 min	116.6 (7.82)	116.5 (6.82)
30 min	114.8 (7.91)	115.9 (5.19)
1 hour	117.2 (10.39)	115.0 (7.30)
2 hours	114.8 (7.59)	115.0 (7.23)
4 hours	115.7 (8.43)	115.3 (5.87)
6 hours	118.3 (5.08)	117.8 (4.97)
8 hours	119.3 (3.03)	118.5 (3.98)
12 hours	119.4 (5.06)	121.0 (4.61)
24 hours	119.1 (3.23)	119.4 (6.05)

Repeated measures ANOVA was used to evaluate the sustained effect of group allocation on diastolic blood pressure across all 14 time points concurrently. The overall between-group F-test was non-significant (p = 0.23), confirming that diastolic blood pressure did not differ systematically between the two epidural drug groups over the study period (Table 11).

**Table 11:** Diastolic blood pressure (mmHg) at different time points between the groups The repeated measures ANOVA was used to evaluate the sustained effect of group allocation on diastolic blood pressure across all 14 time points concurrently. In this two-factor model, the between-subjects factor is drug group--each patient is nested within exactly one group and contributes no data to the other--while the within-subjects factor is time, with each patient providing 14 serial diastolic readings. The model accounts for the fact that repeated measurements within the same patient are correlated, which would otherwise inflate the Type I error rate if multiple t-tests were used alone. The covariance structure of the within-subject observations was modelled using the compound symmetry or Greenhouse-Geisser correction as appropriate. Repeated measures ANOVA: p = 0.23.

Time point	0.0625% bupivacaine + fentanyl (n=20); mean (SD)	0.1% ropivacaine + fentanyl (n=20); mean (SD)
Baseline	78.4 (6.12)	80.2 (7.32)
5 min	75.9 (5.24)	76.7 (6.92)
10 min	70.3 (6.09)	73.7 (6.48)
15 min	68.7 (7.80)	71.8 (7.10)
20 min	69.6 (6.91)	72.6 (5.89)
25 min	72.2 (5.71)	72.7 (7.90)
30 min	69.6 (6.62)	71.6 (5.83)
1 hour	72.0 (7.50)	73.5 (7.36)
2 hours	73.0 (5.43)	73.8 (6.65)
4 hours	72.2 (7.10)	73.1 (5.49)
6 hours	73.5 (4.03)	73.9 (4.58)
8 hours	75.7 (4.17)	77.4 (5.03)
12 hours	77.2 (4.21)	76.8 (5.52)
24 hours	75.4 (4.71)	76.3 (4.96)

Repeated measures ANOVA showed no significant overall between-group difference in mean arterial pressure over time (p = 0.24), indicating comparable mean arterial pressure profiles throughout the study period (Table 12).

**Table 12:** MAP (mmHg) at different time points between the groups The repeated measures ANOVA treated drug group as the between-subjects factor, with 20 patients per group, each randomized to receive either the bupivacaine or ropivacaine epidural regimen. The within-subjects factor comprised the serial MAP measurements across time, with the model estimating how MAP evolves over time within each patient and whether this temporal trajectory differs between the two groups. The group × time interaction term in the model tests whether the rate of change of MAP over time differs between groups, while the main group effect tests whether one group has consistently higher or lower MAP across all time points. MAP: mean arterial pressure Repeated measures ANOVA: p = 0.24.

Time point	0.0625% bupivacaine + fentanyl (n=20); mean (SD)	0.1% ropivacaine + fentanyl (n=20); mean (SD)
Baseline	94.5 (5.22)	96.6 (6.02)
5 min	90.5 (5.52)	92.4 (6.43)
10 min	85.6 (6.41)	89.1 (5.69)
15 min	84.4 (7.12)	87.2 (6.42)
20 min	84.2 (6.95)	87.1 (4.80)
25 min	87.0 (5.38)	87.3 (7.12)
30 min	84.7 (6.05)	86.4 (5.17)
1 hour	87.0 (6.85)	87.3 (6.90)
2 hours	86.9 (4.96)	87.5 (6.31)
4 hours*	86.7 (6.29)	87.2 (5.04)
6 hours*	88.4 (3.73)	88.5 (3.95)
8 hours	90.2 (3.31)	91.1 (3.79)
12 hours	91.3 (3.25)	91.5 (3.91)
24 hours	90.0 (3.93)	90.6 (4.42)

## Discussion

The demographic and obstetric characteristics of the two groups were comparable. There were no statistically significant differences in age, height, weight, body mass index, parity, gestational age, or cervical dilatation at the time of epidural placement.

Maternal hemodynamic stability is a major concern during neuraxial labour analgesia [10,11]. In the present study, heart rate, systolic blood pressure, diastolic blood pressure, and mean arterial pressure remained stable throughout the observation period in both groups. Repeated measure ANOVA demonstrated no significant intergroup differences. Although a mild decline in hemodynamic parameters was observed after epidural administration, values remained within clinically acceptable limits and recovered subsequently without intervention. These findings are consistent with previous studies evaluating low-concentration local anaesthetic-opioid combinations for labour analgesia, which have reported excellent cardiovascular stability due to minimal sympathetic blockade [12-14].

Pain relief was assessed using the VAS. Baseline pain scores were identical in both groups. Following epidural administration, pain scores decreased significantly in both groups, confirming the effectiveness of both regimens. However, Group A achieved superior analgesia during the early phase of labour, with significantly lower VAS scores at 5, 10, and 15 minutes after drug administration. Furthermore, pain scores at 1 and 2 hours remained significantly lower in Group A (Table 3). These findings indicate a more rapid onset and better maintenance of analgesia. The superior analgesic efficacy observed in Group A may be attributed to enhanced sensory blockade and improved spread of the epidural solution, resulting in more effective attenuation of labour pain pathways. Bawdane et al., Chethanananda et al., Gajjar and Patel, Das et al., as well as Shenvi and Jaiswal showed equivalent analgesia in bupivacaine and ropivacaine groups [12-15,18].

Maternal satisfaction is an important outcome measure because it reflects the overall childbirth experience. In the present study, maternal satisfaction was significantly greater in Group A, with a higher proportion of women rating their analgesic experience as excellent. Improved satisfaction likely resulted from the faster onset of analgesia, lower pain scores, and reduced need for rescue doses observed in this group (Table 8). In studies by Bawdane et al., Chethanananda et al., Das et al., Shenvi and Jaiswal, and Clement et al., there was no statistically significant difference in maternal satisfaction between the two groups [12-14,18,19].

The highest sensory block level achieved was comparable between groups, with most parturients attaining a sensory block of T8 (Table 5). This suggests that both regimens ultimately provided an adequate extent of sensory blockade for labour analgesia, although Group A achieved the desired block more rapidly. This was comparable to studies by Chethanananda et al. and Gajjar and Patel [14,15].

The onset of sensory blockade up to the T10 dermatomal level was significantly faster in Group A than in Group B (13.45 ± 3.66 minutes versus 16.0 ± 3.48 minutes) (Table 4). Rapid establishment of analgesia is highly desirable in labouring women because it improves comfort and reduces maternal stress during painful uterine contractions. In studies such as Bawdane et al., Chethanananda et al., and Das et al., the onset of analgesia was similar in both drugs [12-14]. This result is in contrast to that of the study by Shenvi and Jaiswal, where the ropivacaine group had a faster onset of analgesia [18].

An important objective of modern labour epidural analgesia is the preservation of motor function. No motor blockade was observed in either group, as all parturients maintained a Modified Bromage score of 0. The absence of motor blockade is clinically advantageous because it allows maternal mobility, facilitates effective bearing-down efforts during the second stage of labour, and minimises instrumental delivery rates. These findings support the use of dilute local anaesthetic solutions combined with opioids to achieve selective sensory blockade while preserving motor function. Campbell et al. compared 0.08% bupivacaine and fentanyl with 0.08% ropivacaine and fentanyl. Their results indicated that the ropivacaine group is better at preserving the parturient's ability to ambulate than the bupivacaine group [20]. Bawdane et al. had compared bupivacaine 0.1% with fentanyl 2 mcg/mL and ropivacaine 0.1% with fentanyl 2 mcg/mL for labour analgesia. They had observed that the three parturients in the bupivacaine group had a motor block and were delivered using forceps [12].

The mean number of epidural top-ups was lower in Group A than in Group B (Table 6), and the total drug requirement (mL) was also significantly reduced (Table 7). Reduced need for supplemental dosing reflects superior analgesic efficacy and longer duration of action. Lower drug consumption may additionally decrease the risk of dose-related adverse effects and improve overall cost-effectiveness. This is in contrast to the results of the study done by Gajjar and Patel, where the ropivacaine group patients required a smaller number of top-up doses. This may be because of the use of a higher concentration of ropivacaine [15]. In other studies by Bawdane et al., Das et al., and Shenvi and Jaiswal, the number of top-ups required was comparable in both groups [12,13,18]. Other studies have shown that the total drug requirement was similar for both drugs [12-14,21-24].

One of the major concerns regarding epidural labour analgesia is its potential effect on labour progress. In the present study, the duration of both the first and second stages of labour was comparable between groups. These findings indicate that neither regimen adversely affected the normal progression of labour. Contemporary evidence similarly suggests that low-dose epidural techniques do not significantly prolong labour when compared with alternative analgesic strategies [7,10].

The mode of delivery was also similar between groups. Most women delivered vaginally, while five parturients (three in Group A and two in Group B) underwent lower segment cesarean section due to obstetric indications such as non-reassuring fetal heart rate, failure to progress in labour, or cephalopelvic disproportion. The incidence of caesarean delivery did not differ significantly between the groups. These findings support current evidence indicating that well-conducted epidural analgesia does not increase operative delivery rates.

Neonatal outcomes were assessed using APGAR scores at one and five minutes. No significant differences were observed between groups, and all neonates demonstrated satisfactory adaptation after birth. These findings indicate that both epidural regimens were safe for the fetus and did not adversely affect neonatal well-being. No adverse effects were observed in either group. The absence of significant maternal complications further supports the safety profile of both regimens. The use of low concentrations of local anaesthetics with opioid adjuvants likely contributed to the low incidence of complications while maintaining effective analgesia.

Overall, the present study demonstrates that both epidural regimens provide safe and effective labour analgesia. However, Group A offered several clinically relevant advantages, including a faster onset of sensory blockade, superior early analgesia, reduced requirement for supplemental doses, lower total drug consumption, and higher maternal satisfaction, while maintaining comparable maternal hemodynamics, labour outcomes, mode of delivery, neonatal outcomes, and safety profile. These findings suggest that Group A may represent a more effective epidural analgesic regimen for labouring women.

One of the key strengths of this study was the use of epidural analgesia, which ensured effective pain control during labour and contributed to a more comfortable childbirth experience. The administration of low concentrations of local anaesthetic agents helped to decrease total drug consumption while reducing the likelihood of adverse effects, including motor blockade. In addition, participants were closely monitored throughout the study to identify any complications.

Nevertheless, the study had certain limitations. Since it was conducted at a single institution with a relatively small number of participants, the applicability of the findings to larger and more diverse populations may be limited. The effect of epidural bolus injection speed on injection pressure and its potential impact on analgesic outcomes requires further evaluation. Future studies should also explore the long-term implications of labour epidural analgesia, particularly its relationship with chronic postpartum pain and the development of postnatal depression [25].

## Conclusions

Epidural labour analgesia with 0.0625% bupivacaine combined with fentanyl (2 mcg/mL) provides superior analgesic efficacy, faster onset and reduced drug requirements compared to 0.1% ropivacaine with fentanyl (2 mcg/mL), while maintaining a comparable safety profile. These findings support the use of low-concentration bupivacaine-fentanyl combinations as an effective option for labour analgesia, providing excellent pain relief without compromising maternal or neonatal safety. Larger multicentre studies are warranted to validate these results and establish optimal epidural analgesic regimens for routine clinical practice.
